# Cultivating participatory processes in self‐harm app development: A case‐study and working methodology

**DOI:** 10.1002/jcv2.12295

**Published:** 2024-12-11

**Authors:** Camilla M. Babbage, Joanna Lockwood, Lily Roberts, Josimar Mendes, Chris Greenhalgh, Lucy‐Paige Willingham, Emmanuel Wokomah, Rebecca Woodcock, Petr Slovak, Ellen Townsend, the Digital Youth research team, Ellen Townsend, Chris Hollis, Jo Gregory, Elvira Perez Vallejos, Rebecca Woodcock, Peter Fonagy, Louise Arseneault, Sarah Doherty, Lucy‐Paige Willingham, Cathy Creswell, Emily Lloyd, Josimar De Alcantara Mendes, Carolyn Ten Holter, Marina Jirotka, Praveetha Patalay, Yvonne Kelly, Aaron Kandola, Edmund Sonuga‐Barke, Sonia Livingstone, Kasia Kostryka‐Allchorne, Mariya Stoilova, Rory O'Connor, Dorothee Auer, Sieun Lee, Nitish Jawahar, Marianne Etherson, Chris Greenhalgh, Kapil Sayal, Jim Warren, Vajisha Wanniarachchi, Paul Stallard, Charlotte Hall, Mathijs Lucassen, Sally Merry, Karolina Stasiak, Camilla Babbage, Adam Parker, Holly Griffiths, Lily Roberts, Petr Slovak, Amy Jess Williams, Joanna Lockwood

**Affiliations:** ^1^ NIHR MindTech HealthTech Research Centre University of Nottingham Mental Health and Clinical Neurosciences Nottingham UK; ^2^ UKRI Digital Youth University of Nottingham Mental Health and Clinical Neurosciences Nottingham UK; ^3^ Department of Computer Science University of Oxford Responsible Technology Institute Oxford UK; ^4^ School of Computer Science University of Nottingham Nottingham UK; ^5^ Kings College London London UK; ^6^ School of Psychology University of Nottingham Nottingham UK

**Keywords:** card‐sort task for self‐harm, coproduction, digital interventions, PPI, self‐harm, young people

## Abstract

**Background:**

Self‐harm and suicide related behaviours are increasing in young people, and clinical support is not adequately meeting needs. Improved approaches to assessment and the clinical management of self‐harm will result from codesign processes and include greater shared decision‐making between young people and practitioners. The CaTS‐App (an adapted digital version of the existing Card‐Sort Task for Self‐harm research tool) aims to facilitate a collaborative understanding of adolescent self‐harm and support decision‐making within clinical settings. The codevelopment of a digital, clinical tool which meets the needs of multiple stakeholders requires careful consideration.

**Methods:**

We present a case‐study describing the participatory aspects of the development of the CaTS‐App, which included comprehensive patient involvement, research activities and coproduction with diverse young people aged 17–24 with lived experience of self‐harm. We share our processes and activities to deliver safe, engaging, sustainable, ethical and responsible participatory practice and co‐created knowledge, in the codevelopment of the CaTS‐App.

**Results:**

Activities spanned a 48‐month period in both face‐to‐face and online settings. Example processes and activities are provided in narrative, tabular and diagrammatic form, alongside discussion of the rationale for choices made. A summary methodology is also shared to stimulate continued discussion and development of participatory approaches in digital mental health.

**Conclusions:**

The paper contributes important insight and practical detail for the delivery of genuine participatory processes in digital mental health development when working with a population who may be considered vulnerable.

## Graphical abstract

We discuss a case‐study and working methodology for meaningful, safe and responsible involvement in the early Planning and Discovery phase of the development of a novel assessment and intervention app for self‐harm—the CaTS‐App This paper contributes to important discussions about the process and value of centering and evaluating participatory processes in the development of digital mental health interventions for self‐harm.

**Key points**

- Digital mental health interventions for self‐harm codeveloped with young people with lived experience may lead to better outcomes, but often these young people are excluded from involvement opportunities
- We discuss a case‐study and working methodology for meaningful, safe and responsible involvement in the early Planning and Discovery phase of the development of a novel assessment and intervention app for self‐harm—the CaTS‐App
- We reflect on activities to deliver involvement in line with the 5 key priorities, drawing from existing and emerging models and principles from the fields of User Centred Design (UCD), Human Computer Interaction (HCI) and Responsible Research and Innovation (RRI). This paper contributes to important discussions about the process and value of centering and evaluating participatory processes in the development of digital mental health interventions for self‐harm

## INTRODUCTION

Rates of self‐harm and suicide related behaviours have shown an upward trend in recent years and remain a concerning public health priority in the UK (Geulayov et al., 2018; Health and Social Care Committee, 2021; McManus et al., 2019; Nuffield Trust, 2023). Self‐harm is defined here as any act of self‐injury or self‐poisoning, irrespective of motive or suicidal intent (Hawton et al., 2003). There have been several studies that document the challenges in the identification, management, and effective treatment of self‐harm in young people. Current clinical services are insufficient, not always effective, and viewed negatively by young people (Uddin et al., 2023). For myriad reasons formal help is more often not sought at all. Improving how we understand and meet the needs of young people who self‐harm is critical given that the behaviour is an indication of significant distress, and a substantial risk factor for completed suicide.

### Improving the joint assessment and management of self‐harm

Recommendations to improve our approaches to the assessment and management of self‐harm in young people more recently have prioritised an increase in shared discussion and decision making between a young person and practitioner (National Institute for Health and Care Excellence, 2022; University of Manchester, 2024) and recognise that by including youth in a collaborative discussion to understand and identify needs and plan treatment choices, outcomes are likely to be improved (Hasking et al., 2023; Lewis & Hasking, 2019; Quinlivan et al., 2022). There is also recognition that there needs to be flexibility about when, where and with whom these collaborative discussions best take place, and that this might be outside of a traditional clinical service setting in a community or online space (National Institute for Health and Care Excellence, 2022; Robinson, Bailey, et al., 2018; University of Manchester, 2024). Recent guidance provides for a range of frontline settings including education/social care/third sector spaces to talk collaboratively with young people about the support they need (National Institute for Health and Care Excellence, 2022). Development of new or adapted tools and resources and ways of working to better enable practitioners across settings to meet the challenge of supporting collaborative and shared understanding and decision making are needed. It is critical however that any approaches to designing and developing these essential tools and ways of working are also a collaborative, shared process, coproduced and codesigned with those who will be using them (Thabrew et al., 2018).

### Potential of CaTS‐App to support understanding and collaborative decision‐making

The CaTS is a paper‐based research tool which uses a card‐sorting methodology to allow a young person to map how different factors associated with an episode of self‐harm play out along a timeline (Townsend et al., 2016). CaTS facilitates communication of the process and meaning behind a young person's self‐harm (Lockwood et al., 2018; Townsend et al., 2016). A pilot online version of the CaTS designed to replicate the paper‐based version and which can be completed remotely, was successful in indicating salient factors leading up to and following self‐harm in young people and adults (Lockwood et al., 2023). We believe CaTS has potential to support shared understanding and collaborative decision‐making in a clinical setting by scaffolding discussion around the interplay between temporal elements and multiple complex factors relating to self‐harm, and offering a structure to pinpoint individual ‘warning signs’ and patterns of behaviour and identify targeted and time‐specific points for intervention (Lockwood et al., 2023). Furthermore, early research stemming from the Digital Youth programme of work has highlighted the appetite for a digital application of the CaTS (CaTS‐App) from both young people and professional stakeholders (Lockwood et al., 2024).

### A tool *codesigned and co‐created* with young people with lived experience?

Despite well‐established guiding principles which set out that children and young people have a right to have a say in matters which affect them (Lundy, 2007) and that this is at the core of their best interests (Mendes & Ormerod, 2019; National Institute for Health and Care Research, 2021), there are undoubted challenges in providing sufficient opportunities for youth to be heard in a complex and fluid mental healthcare landscape. Despite a growing evidence base indicating the value of providing research opportunities for these voices to be heard, it can be especially difficult to involve children and young people who may have perceived (by both clinicians and researchers) additional vulnerability due to sensitive and more complex mental health difficulties including self‐harm and suicide‐related thoughts and behaviours (Dazzi et al., 2014; Lockwood et al., 2018). Indeed, a systematic review of nearly 100 suicide prevention studies reported no codevelopment (also termed public and patient involvement, PPI) with young people in either the design of or research into the interventions (Robinson, Bailey, et al., 2018). Nonetheless, a recent scoping review suggests improvements in the area with the identification of 22 interventions across adults and young people, spanning different self‐harm intervention points with some level of involvement (Wright et al., 2023). However, few of the studies were found to involve codesigners throughout the research process for example, in grant processes, protocol developmental, intervention and outcome selection, and most studies focussed on initial design activities for the intervention. To aid future codesign, it is recommended that future publications push for transparency, including details on *how* activities are completed (Russell‐Bennett et al., 2023; Wright et al., 2023).

Benchmarks for how to involve young people safely and effectively in the development of self‐harm and suicide resources are set by recent studies that have used participatory approaches for talking about suicide and self‐harm online, and which have provided details of the participatory steps (Michail et al., 2023; Robinson et al., 2023; Robinson, Hill, et al., 2018; Thorn et al., 2020; Wright et al., 2023). Simultaneously, these authors recognise there is still need for continued efforts to ensure meaningful involvement (e.g. increased diversity, transparency in reporting, wider and more sustained involvement at different stages of the development process, greater stakeholder involvement). In terms of youth participatory approaches in the development of *digital* mental health resources and interventions for self‐harm and suicide related behaviour, it is noticeable that very few mental health apps have been specifically designed and developed *for and with* young people, with some exceptions (Grist et al., 2018; Hetrick et al., 2018; Owens & Charles, 2016; Stallard et al., 2016). Recent reviews have called for digital interventions for self‐harm to be codeveloped and coproduced in collaboration with future users, including those who have lived experience, recognising that this is not simply collaboration for its own sake but in order to lead to interventions and resources that are more likely to meet real world needs and be acceptable to young people (Arshad et al., 2020; Cliffe & Stallard, 2023; Kennard et al., 2018; Kruzan et al., 2022; Lewis & Hasking, 2019; Michail et al., 2023; Robinson, Bailey, et al., 2018). Yet knowing *how* to optimally undertake coproduction and PPI processes with young people with lived experience and planning for this process can feel daunting.

We welcome moves towards more detailed sharing of the methods adopted by researchers undertaking participatory work with youth in the self‐harm and suicide field sometimes referred to as user‐centred design (UCD) or person‐based approaches (PBA), which aim to centre end‐users in the development of new technologies (Bevan Jones et al., 2018; Kruzan et al., 2021; Yardley et al., 2015). Pioneering work by Kruzan et al. (2021) shared methods of working with individuals with lived experience of self‐harm within a user‐centred design approach, documenting formative and evolving processes (i.e., elicitation, design, evaluation) of a suicide prevention intervention app. In addition, Gan et al. (2023) detailed the codevelopment processes and activities they employed to promote engagement with a smartphone app to help young people manage suicidal thoughts, and Han et al. (2019) shared first steps in a participatory approach to manage suicide ideation. Alongside detailed guidance (Kruzan et al., 2021), shared procedures facilitate researchers to plan their own codevelopment and can provide a helpful starting point for understanding and delivering what works.

### Drawing from existing models, principles and frameworks

So, how should we proceed with the development of the proposed CaTS‐App which aims to support collaborative assessment and decision making for self‐harm support and has diverse requirements including: meeting multiple stakeholder needs (young people, clinicians, practitioners, professionals) delivering a structured evidence‐based task (card‐sort task for self‐harm) within the parameters of a new digital version; and implementation in diverse and complex settings (clinical, community based)?

Recent studies have provided broader direction for researchers developing interventions in this field. Čuš et al. (2021) recognised the diversity of factors that influence engagement with an app, recommending collaboration among people with lived experience, mental health professionals, and professionals in Human Computer interaction (HCI) and implementation science. Cross‐discipline collaboration is also emphasised in other digital mental health development literature (Bevan Jones et al., 2018; Pearce et al., 2022). We can draw on existing knowledge and frameworks from established UCD and implementation science fields to help frame our approach. For example, continuing to centre the young person in the development of the intervention, the person‐based approach also considers stakeholders as an integral part of the process (Yardley et al., 2015). In early intervention design, the PBA seeks to identify facilitators, barriers and contextual issues relevant to intervention implementation. This leads to the development of guiding principles that support understanding of how a tool can address the needs of the population it is being used with (Morrison et al., 2018). Similarly, in complex intervention design guidelines from the Medical Research Council, early steps require a contextual understanding of how an intervention would be useful and iterative design refinements are used to ensure acceptable and feasible implementation at later stages (Skivington et al., 2018). These HCI and implementation approaches are complementary to the approaches we would take within Medicine.

In addition, emerging work in the field of Responsible Research and Innovation (RRI) not only recommends the engagement of future users and key stakeholders in digital mental health development, but mandates the outlining of strategies to anticipate and mitigate potential negative or adverse impacts and outcomes from digital development (Inglesant et al., 2016; Jirotka et al., 2017). We are not aware of existing research exploring the application of RRI within self‐harm DMHI development and consider such an approach an integral component for safe and responsible involvement with young people who may be high‐risk.

### Aim of this paper

The aim of this paper is primarily to share the approach and activities we have developed to deliver PPI and coproduction with young people with lived experience in the participatory (Planning and Discovery, Design and Development) phases of the CaTS‐App development;

Key priorities of our approach include.

- Meaningful, safe, responsible and engaging collaboration with young people who can be considered high‐risk
- Early and sustained involvement, including young people in initial planning and discovery phases
- Inclusion of diverse and marginalised voices
- Putting in place measures to chart how the process of participation is contributing to outcomes and output, and informing subsequent stages
- Guided by existing models/approaches/principles relevant to overall experience design

Additionally, we will share a working methodology for the development of the CaTS‐App which draws from existing and emerging principles and knowledge across self‐harm and mental healthcare, PPI, RRI and HCI/implementation science relevant to experience design.

### Overview of Digital Youth programme

**FIGURE 1:**
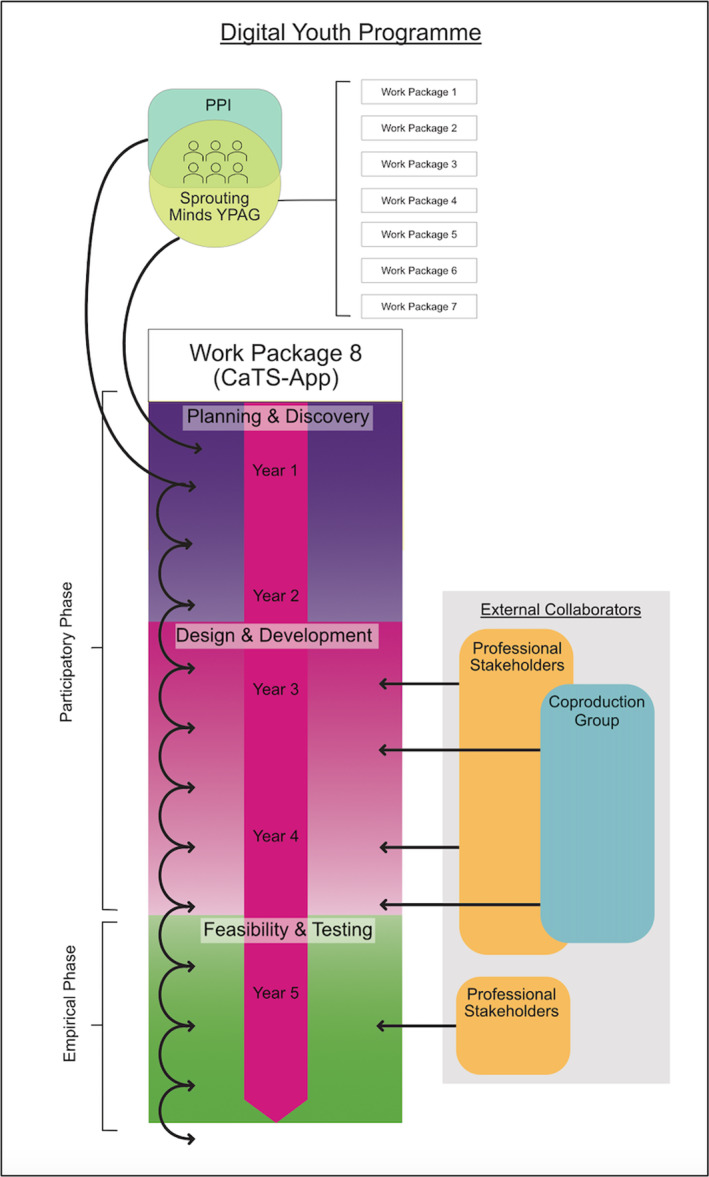
This figure provides an overview of the CaTS‐App Work Package (WP8), within the structure of Digital Youth. Young people in the CaTS‐App PPI group are also members of the Sprouting Minds YPAG, offering young people the possibility of being involved across the Programme's Work Packages enabling young people to get involved in research in a flexible manner at different levels of involvement, whilst creating sustained involvement and training by Sprouting Minds. The image also provides an overview of the different phases of the CaTS‐App and the stages within these phases, plus how and when external stakeholders were involved.

An overview of the Digital Youth programme can be seen in Figure 1. Digital Youth is a 5‐year interdisciplinary UKRI funded research programme focussed on understanding complex risks and opportunities for mental health associated with young people's engagement with the digital world. Digital Youth comprises eight work packages, one of which is the development of the CaTS‐App. The programme has a PPI Manager and a PPI Involvement and Engagement Manager to provide support to Sprouting Minds throughout the programme. An aim of Digital Youth is to imbed involvement with young people across the research programme, supported by two co‐applicants who are young people with lived experience of mental health difficulties who sit in leadership roles and who were instrumental in obtaining funding for the programme. These young people are the co‐chairs of Digital Youth's Young Person's Advisory Group (YPAG), Sprouting Minds, a youth‐led, inclusive group of young people (nicknamed ‘Sprouts’), aged 16–25 who have lived experienced of mental health difficulties. Young people within Sprouting Minds are part of a generic YPAG group with co‐chair led core meetings and can also commit to being a PPI member within any of the work packages where they will join specific activities in addition to the core YPAG. This structure enables young people to get involved in PPI in a flexible manner, feeding back more generally to research projects as part of the core YPAG, or at a more granular level as part of a specific work package remit.

From hereon, young people involved in the core Sprouting Minds YPAG will be referred to as ‘Sprouting Minds’, and those who are also involved in CaTS‐App will be referred to as the PPI group to make a distinction between levels of involvement. Further, young people involved in participatory activities who are external to Sprouting Minds will be referenced as the ‘Coproduction Group’. This structure is outlined in Figure 1.

## METHODS

### Case‐study: Developing CaTS‐App with young people—Early phase PPI and coproduction with young people

The overall aim of the CaTS‐App project is to codevelop and test the feasibility of a digital version of the card‐sort task for self‐harm. In line with formative UCD or person‐based processes in DMHI development (Bevan Jones et al., 2018; Kruzan et al., 2021), the project spans three phases: Planning and Discovery; Design and Development; and Feasibility and Testing). In this case‐study we focus on the first two phases (the participatory phases of the CaTS‐App development), taking the original research tool, CaTS (Townsend et al., 2016) and working alongside young people and professional stakeholders to explore the need for, content and design of a potential digital version of CaTS, referred to as “CaTS‐App”, ahead of empirical work. In our Planning work we sought to robustly embed lived experience expertise into the development process from the outset via Sprouting Minds and PPI groups. Our Discovery work sought to understand needs, preferences, contextual factors of key stakeholders (via interviews/focus groups/surveys) with PPI involvement and coproduction workshops. Our Design and Development Phase included a further round of coproduction workshops with PPI involvement leading to a prototype of the CaTS‐App.

Here we share the approach and activities we have developed to deliver involvement and coproduction with young people with lived experience throughout the participatory phases. A description of each activity can be seen in Table 1. An overview of the participatory work for the CaTS‐App within the Digital Youth programme is shown in Figure 1.

**TABLE 1:** Description of activities associated with Sprouting Minds and Coproduction.

Activity	Description of activity	Sprouting Minds (YPAG)	CaTS‐App PPI	Coproduction Group
**Project posters**	An informative recruitment poster inviting PPI to the project, giving an overview of the purpose and details on what PPI will look like	⚫		
**Project timelines**	A pictorial timeline with project overview		⚫	
**Introductions**	Name, photo and contact details of research team to help identify who is responsible for what		⚫	⚫
**Agenda**	An overview of the upcoming meeting		⚫	⚫
**Shared online libraries**	An online location where PPI can access documents relating to involvement		⚫	
**Group agreement**	Designed with the group, the agreement captures how the group wants to look, feel and work together and is signed by all PPI members and researchers		⚫	
**Collaborative platforms**	Free platforms to collaborate that support accessible and flexible involvement		⚫	⚫
**Mental health inclusive language**	A meeting to agree on terms that will be used surrounding mental health and self‐harm		⚫	
**Visual identity**	The use of graphics and/or logos to create a sense of identity with the group		⚫	
**Wellness plan**	A document highlighting a care plan for a PPI member, including emergency contact details, self‐care strategies, sources of support and methods for taking a break from the project or a session		⚫	⚫
**Resources and support**	A signposting document with crisis hotlines and self‐harm resources		⚫	⚫
**Triggering triangles**	A triangle placed in the top corner of documents or activities to highlight they could be triggering		⚫	⚫
**VAS scale**	A scale used to prompt reflection on mood change at the start and end of PPI sessions		⚫	⚫
**Fun activities**	Mood boosting interactive activities to conclude PPI sessions, lasting 5 min		⚫	⚫
**End of session check‐in**	Researchers remain in the meeting at the end of a session so that PPI members can talk to them		⚫	⚫
**Debriefs**	A succinct write up of changes and actions points resulting from all PPI involvement		⚫	
**Updates**	Monthly project updates to inform PPI members of changes to the project		⚫	
**Meeting trackers**	A spreadsheet documenting the date, length and time spent on involvement activities to support claims		⚫	
**Feedback**	Inviting PPI groups to reflect and give feedback on being involved as an involvement member	⚫	⚫	
**Key learnings**	Young people are invited to review the key learnings and make changes		⚫	⚫
**Advertisement**	An informative recruitment poster inviting young people to be a part of the coproduction workshops with a link to register interest			⚫
**Project flow chart**	The steps of coproduction are depicted as images with a sentence explaining the process			⚫
**What to expect**	A text‐based flow chart which aimed to give a written concise summary for each stage of the project			⚫
**Information sheet**	Part of the information pack, the information sheet contains all the necessary information about involvement processes			⚫
**Ways of working**	Designed with PPI, the ways of working asks young people to agree to working safely and collaboratively together			⚫
**Your experience**	A document was given to prepare young people for a conversation around the young person's experience of self‐harm, to ensure the young person wasn't currently self‐harming or suicidal			⚫
**Initial phone call**	A phone call between interested young people and a researcher to complete an eligibility check, a wellbeing check and taking verbal consent			⚫
**Online agreement**	Online‐form asking young people to agree to the ways of working, capture accessibility requirements, availability and emergency contact information			⚫
**Confirmation notice**	An invite to the workshops, explaining how to access the event, what to expect from it and wellness prompts including signposting and wellness plan reminders			⚫
**Pre‐workshop check‐in**	A quick phone call to check self‐harm/suicide status within 48 h of upcoming workshop			⚫
**Online VAS form**	An online form at the start and end of sessions to monitor young person's mood change with the VAS			⚫
**In‐session support**	Provision of clinical support via an external provider who were available in breakout rooms during sessions			⚫
**Feedback forms**	Online forms collecting feedback on likes or dislikes, if they felt distressed or required further support during the workshops			⚫

### Planning and Discovery phase

#### Sprouting Minds YPAG

The Planning and Discovery phase began with collaboration between the research team and co‐chairs from Sprouting Minds to identify a sub‐group of PPI members who were interested in the CaTS‐App project. A project poster detailing what to expect from the CaTS‐App work package was created and presented to Sprouting Minds to help recruit a PPI subgroup focussed on CaTS. Once in place we collaborated with our PPI group to plan the Discovery stage which included stakeholder research with practitioners and coproduction workshops with young people external to Sprouting Minds to gain foundational understanding of stakeholder views on the CaTS‐App. PPI members were invited to regular online meetings where we planned the design of the coproduction workshops, recruitment, the onboarding process, workshop structure, analysis, and dissemination plans for the coproduction work. The planning phase also required consideration of how key RRI values are relevant to the CaTS‐App and the approaches needed to address these values (Table 2).

**TABLE 2:** RRI table.

Key RRI values	What is its relevance to CaTS‐App?	How it was/is being addressed throughout the development process?
The development of the new technology, product or intervention is socially desirable and based on the public interest (Inglesant et al., 2016)	CaTS‐App aims to fill a gap in robust interventions for self‐harming among young people, particularly those delivered through online platforms	Workshops with self‐harming people and other key stakeholders (clinicians, practitioners, service managers) to discuss the tool development and its scope
Stimulation of creativity in science and innovation (Inglesant et al., 2016)	To foster adherence among young people, CaTS‐App must “speak their language” and mirror their world and experiences	Workshops with self‐harming people to coproduce and codesign the tool
Facilitation of reflexive and inclusive processes (Eden et al., 2014)	To ensure a legitimate involvement process in which young people are truly heard and acknowledged	Involvement of Sprouting Minds
		Workshops with self‐harming people
Transparency, sustainability and accountability (Eden et al., 2014; Von Schomberg, 2013)	To secure a truly responsible and participative approach throughout the development process	Involvement of Sprouting Minds
		Workshops with self‐harming people
Involvement of a broad set of diverse stakeholders (Van den Hoven, 2021)	CaTS‐App will assist psychosocial interventions involving self‐harming young people and clinicians/practitioners	Workshops with self‐harming people
		Conducting surveys, interviews and focus groups clinicians, practitioners, service managers to gather feedback on the tool and the implementation process at CAMHS
Involvement of consumers and end‐users early in the process (Porcari et al., 2016)	To ensure a legitimate involvement process in which young people are truly heard and acknowledged	Involvement of Sprouting Minds
		Workshops with self‐harming people
Anticipation of negative, adverse or unintended impacts and outcomes (Owen & Pansera, 2019)	To foresee any negative or unintended impacts during the development process and future implementation	Workshops with self‐harming people
		VAS scale, end of session check‐in, feedback
Mitigation of negative, adverse or unintended impacts and outcomes (Jirotka et al., 2017)	To ensure our ‘duty of care’ and young people's best interests	Triggering triangles, VAS scale, end of session check‐in, feedback

#### CaTS‐App PPI

We recruited five PPI members to join our project. Those completing demographic information (*n* = 3, 60%) were aged between 21 and 26 years (mean = 24 years), all identified as female, and all were heterosexual, presented in Table 3. One declared living with a long‐term mental health condition, and one declared living with a long‐term physical health condition. One young person was employed, another was unable to work, and the other was looking for work. Educational achievement varied from no GCSEs to undergraduate degrees at higher education level. All three young people were from minority ethnic groups including Asian and multiple ethnicities.

**TABLE 3:** Coproduction workshops demography.

First Workshops				
		Demography		
	Total	Age	Gender	Ethnicity
Workshop 1 (online)	*N* = 3	19–22 (mean = 20.5)	Female = 2 Male = 1	White British = 2
Workshop 1 (in‐person)	*N* = 6	17–24 (mean = 19.2)	Female = 5 Male = 1	White British = 3 Asian Korean = 1 Pakistani British = 1
Workshop 2	*N* = 6	17–24 (mean = 20.4)	Female = 5 Male = 1	White British = 3 Asian Korean = 1 Pakistani British = 1
Workshop 3	*N* = 7	18–22 (mean = 20.2)	Female = 4 Male = 3	White British = 4 Asian Korean = 1 Black African = 1

Second Workshops				
		Demography		
	Total	Age	Gender	Ethnicity
Workshop 1	*N* = 7	18–25 (mean = 20.1)	Female = 4 Male = 3	White British = 4 Black British = 1 Asian Korean = 1 Black African = 1
Workshop 2	*N* = 6	18–25 (mean = 20.3)	Female = 3 Male = 1	White British = 4 Asian Korean = 1 Black African = 1
Workshop 3	*N* = 5	18–25 (mean = 21.0)	Female = 3 Male = 1	White British = 3 Asian Korean = 1 Black African = 1

Being involved in the project required a commitment to meet online, approximately once every 8 weeks, and included occasional offline tasks or individual online meetings with the research team. Since January 2022, we have held 17 online sessions of group work, three offline tasks and two individual meetings with the PPI group as part of Planning and Discovery phase.

**FIGURE 2:**
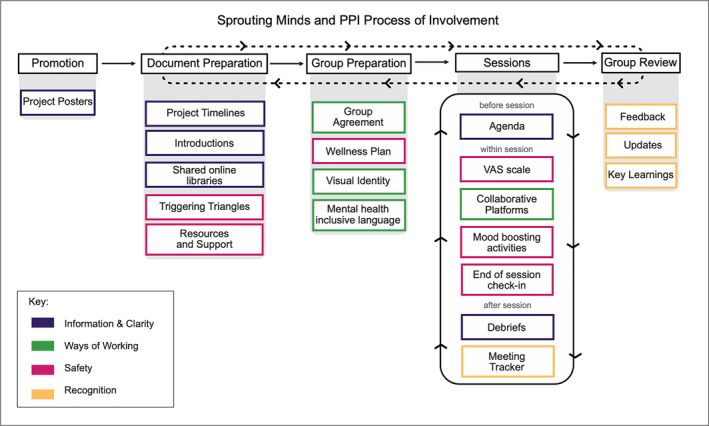
This figure shows the researcher's process of involvement for PPI members. The process highlights the activities that occur before, within and after PPI sessions (see Table 1 for a description of the activities) and how these processes relate to the key objective they support (see Table 4). The process of involvement flows from left to right, some including cyclical activities that repeat.

The process of involvement for researchers when involving young people as PPI members in the CaTS‐App is presented in Figure 2. Ahead of initial meetings with the PPI group, document preparation included creating an overview of the project and to develop processes to support PPI members' wellness during the project. Time was spent designing friendly materials that were easy to digest and aesthetically pleasing, with the addition of logos, colourful graphics, and use of features such as ‘triggering triangles’. These documents were held on shared libraries that could be easily accessed within and between sessions.

**FIGURE 3:**
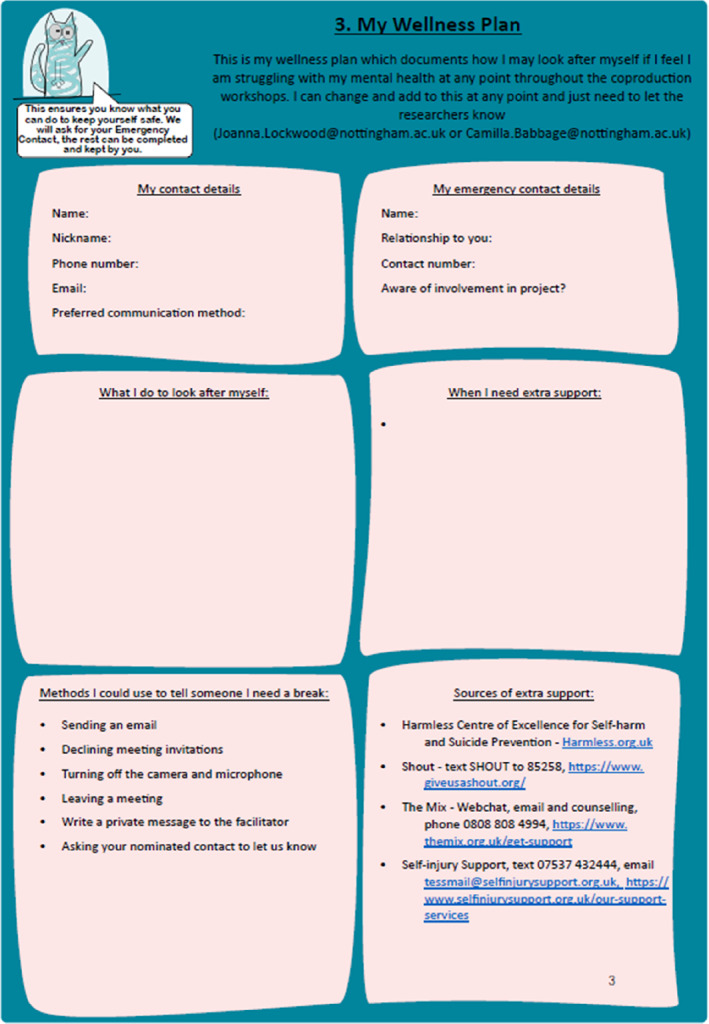
This figure shows the wellness plan. This is a document highlighting a care plan for a PPI member, including emergency contact details, self‐care strategies, sources of support and methods for taking a break from the project or a session.

Preparations with individual PPI members included the development of a wellness plan (Figure 3) in an online one‐to‐one meeting between the researchers and the PPI member to discuss their wellbeing. Together, we considered how a young person would support themselves, or receive support, if their mental health was to deteriorate during the project. The conversation was also used to develop rapport, with an opportunity to explore any wishes the young person had from being involved in the project such as requirements that could be used to support their involvement or personal development opportunities. A personalised wellness plan was created and sent to young people after the meeting and securely stored by the research team.

To support the PPI group dynamic, we developed a group agreement through an open and honest discussion with the PPI members about how we, PPI members and researchers, wanted the group to look and feel. This included practical implications such as when, where, and how often we would meet which was usually in the evening, mid‐week and online via Zoom. Once finalised, this was distributed and all group members, including researchers, were asked to sign the agreement which was reshared with all members. To further support an inclusive group, we ran an online session covering definitions of self‐harm and considering the language we would use together. Such activities would take place using online collaborative platforms that enabled young people to contribute in different ways, for example, through talking, using chat functions or adding comments directly to the platform.

Together with our PPI members we codeveloped objectives for the PPI work to support safe, meaningful, and responsible collaboration when working on a complex, sensitive and long‐term project. We determined four key objectives: information and clarity; ways of working; safety plans; and recognition (see Table 4). We mapped subsequent activities and processes according to these four objectives. So that young people felt informed an agenda was always sent ahead of time so that PPI members could prepare for sessions and get involved offline if it was not possible for them to attend the meeting. In addition, succinct debriefs would be documented and stored in an accessible location. Sessions were tracked on a meeting tracker and young people reminded to use this to support their renumeration, in line with objectives for young people to be recognised. To support wellbeing, sessions were designed to be interactive and engaging for example, with the use of GIFs to support explanations and with the researchers bringing a ‘fun’ energy to sessions. Sessions would end with mood boosting activities such as sharing images of your pets, or playing bingo, and researchers would remain on the call up to 5 minutes after the meeting in case debriefs were required. For safety and safeguarding, a risk protocol was agreed as a group. This included being transparent about what would be involved in terms of how we would ensure safety and mitigate risk, such as clarity around decision making processes for example, in what scenario would we contact emergency contacts. To review PPI members' experience within the group, there were formal and informal opportunities for feedback via anonymous online forms and through group conversations. Core meetings with Sprouting Minds co‐chairs also offered a place to talk to other research team members about their experience, which would be fed back anonymously.

**TABLE 4:** Four objectives of PPI.

Objective	How these objectives support safe meaningful and responsible collaboration
Information and Clarity	Supporting young people's understanding is important as we require young people to be fully informed to be able to share their genuine insights and knowing what to expect also gives young people an opportunity to decide if the activity is appropriate for them
Ways of working	Considering how young people ‘feel’ as part of the group to ensure involvement is accessible and feels collaborative to the PPI members and making sure everyone feels included
Safety	Due to the sensitive nature of the project especially for young people with lived experience who may be vulnerable, attention was given for how we might support young people and prevent them from becoming overwhelmed and supporting their involvement
Recognition	Involving young people includes ensuing they are, and feel, recognised for their contributions to and can also be used as a way to support PPI members continued involvement, particularly when involved as long‐term research projects wax and wane

Our process of PPI involvement can be considered cyclical as the activities completed during sessions are repeated, supporting PPI member's familiarity with what to expect. The documents or activities within document and group preparations are updated as required, and the group review activities were completed when fewer PPI sessions were taking place or as part of a yearly review.

The PPI members will continue to be involved in the project and will be invited to the later stages of the process, including analysis and development of Key Learnings.

#### Coproduction Group workshops

A first round of coproduction workshops were held with the aims of gathering young people's initial thoughts on the CaTS task, exploring features that might be useful in an app version of the CaTS and finally exploring what might motivate and deter use of a digital version of the CaTS.

**FIGURE 4:**
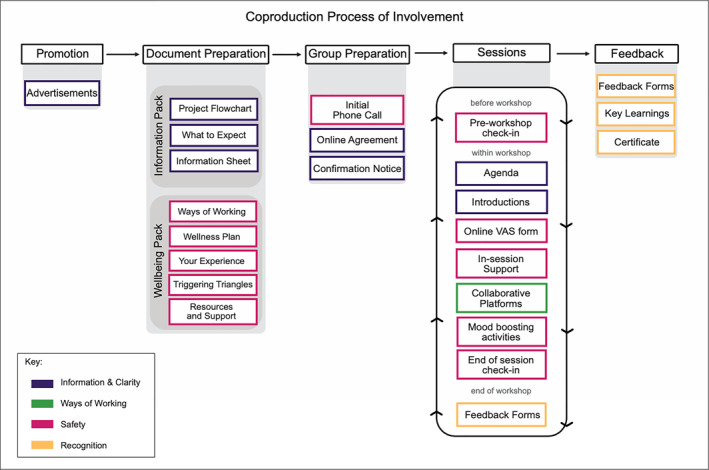
This figure shows the researcher's process of involvement for coproduction. The process highlights the activities included when young people are involved in the coproduction workshops (see Table 1 for a description of the activities). The process flows from top‐left to bottom‐right, with the coproduction workshops repeating activities within each session. Each activity is highlighted in the colour of the key objective it supports, see Table 4.

Young people were invited to three workshops over three weeks of Summer 2023 held across weekends and weekday evenings. The first workshop was offered online and in‐person, young people could select to attend either. The in‐person workshop was 3‐h, held in Nottingham city centre in a collaboratively designed workspace with food and travel provided. The subsequent two workshops were online, lasting 2‐h via Microsoft Teams and utilising Miro as a virtual whiteboard for activities. Applying our four objectives (Table 4): Information and clarity; ways of working; safety; and representation, we have documented the processes used to support young people's involvement during this coproduction. See Figure 4 for an overview.

Eleven young people (8 female, 3 male) took part, aged 17–24 (mean = 19.4 years). All had personal experience with self‐harm, but young people currently self‐harming (within 2 weeks) or with a recent history of suicidal ideation (within 6 months) were advised the workshops would not be appropriate for them.

**FIGURE 5:**
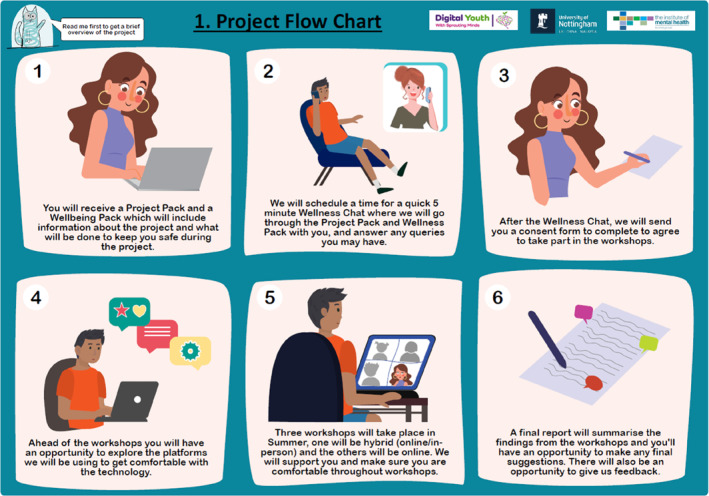
This figures includes the project flow chart. The steps of involvement for the Coproduction Group are depicted as images with a sentence explaining the process.

Advertisements to get involved in the workshops were displayed through online and offline platforms including social media, local youth groups, wellbeing/mental health groups, school settings and waiting rooms in clinical settings. Recruitment via parents was also utilised. On seeing the advertisement, young people registered their interest online and were contacted with an information pack. The information pack utilised a step‐level approach, so each document gave further information as illustration or text. Examples of the project flow can be seen in Figure 5. An initial phone call was scheduled between the young person and researcher, ahead of which a wellbeing pack was sent to give an overview of what to expect during the phone call (also outlined in Figure 5). Researchers would use this phone call to confirm the inclusion criteria and potentially triggering nature of the workshops, but also aimed to convey enthusiasm for the project and thank young people for their interest.

**FIGURE 6:**
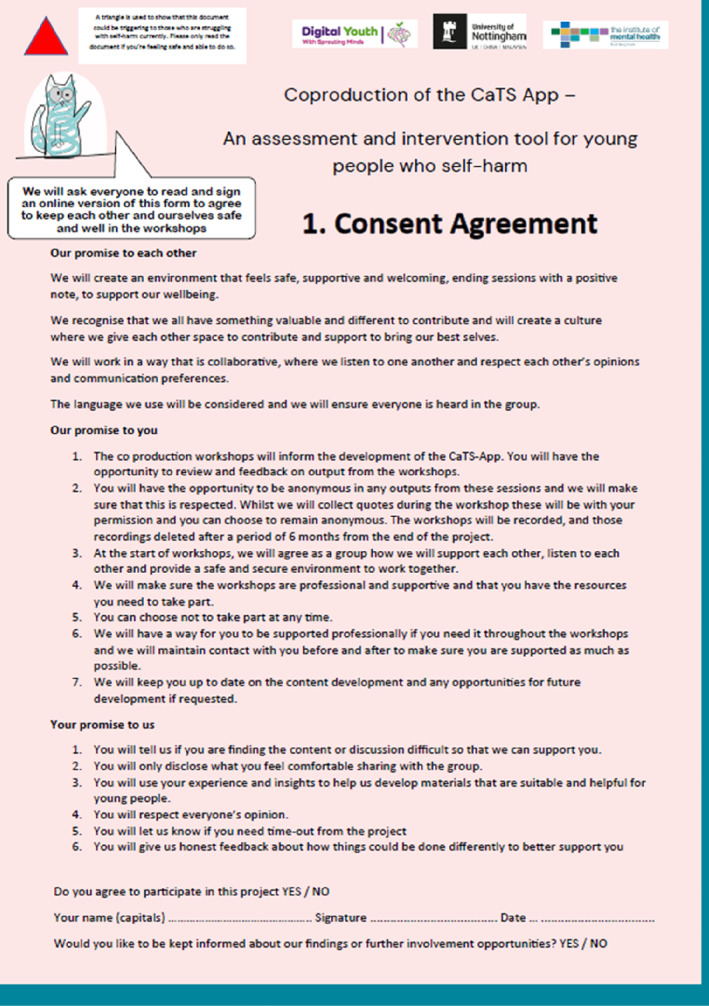
This figure contains the ways of working for the Coproduction Group. This was designed with PPI, and asks young people in the Coproduction Group to agree to working safely and collaboratively together.

**FIGURE 7:**
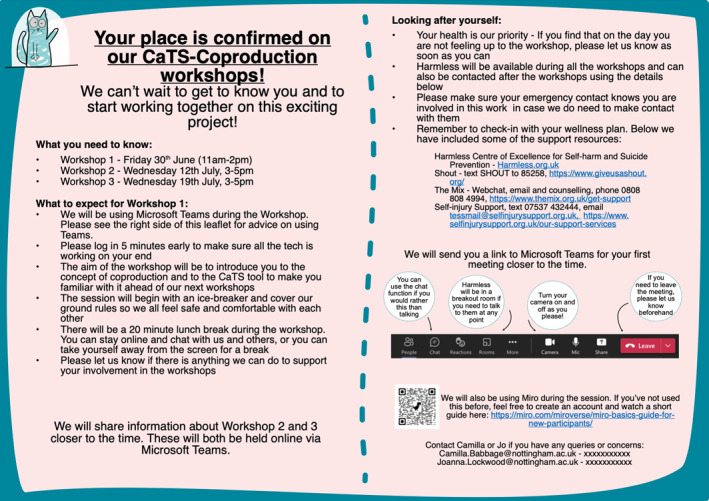
This figure is of the confirmation notice, which is given to young people taking part in the coproduction workshops to explain how to access the event, what to expect from it and wellness prompts including signposting and wellness plan reminders.

Those still wishing to be involved would be asked to agree to the group's Ways of Working‐form (Figure 6) via an Online Agreement‐Form. This was based on the PPI Group Agreement which highlights young people's responsibility for self‐care and respect of one another. To further support safety, between workshops, where there had been no contact with young people within 48‐h of an upcoming workshop, a pre‐workshop check‐in phone call was scheduled to ensure the young person was still in a position to take part. Ahead of the workshops, young people would be sent a Confirmation Notice as illustrated in Figure 7.

**FIGURE 8:**
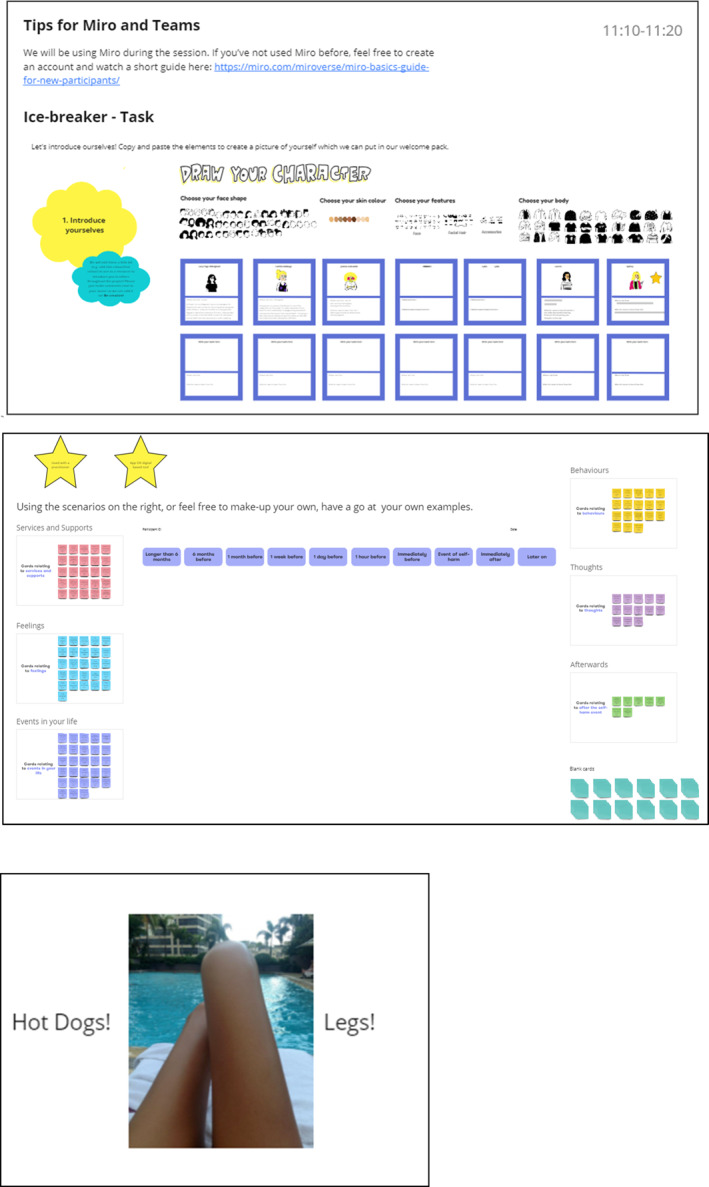
This figure gives example of some of the Miro board features. Miro, a virtual collaborative workboard, was used for all of the coproduction workshops as a way to complete activities.

Sessions followed a similar structure, including technical support, introductions, housekeeping (reminder of the ways of working, in‐session support details and the agenda), the online VAS form, session activities, a break and workshop conclusions (energy booster, online VAS, feedback). Each section had approximate timings to allow young people to skip and return to certain activities if desired. Whilst sessions were guided by the research team, further instructions were available on the board for any information that may have been missed. An idea of what this looked like can be seen in Figure 8.

An introduction activity included an icebreaker where young people could design and name their avatar on the Miro. We revisited this in all subsequent sessions enabling us to make edits and add embellishments to the avatar. Breaks during the session included fun activities, such as ‘hook the question’ a fishing game which selected an ice‐breaker question, and creative activities such as mood boarding.

Safety was supported through the completion of the VAS at the start and end of workshops, and workshops would always end with a mood boosting activity for example, voting on whether a photo was a pair of legs or a pair of hot dogs or asking people to guess the percentage related to a funny fact for example, ‘How many people in the UK like pineapple on pizza?’ In‐session support was also available, including access to Harmless CIC, a Nottingham‐based user‐led community interest company who provide support for people and families with self‐harm and suicide. A Harmless Counsellor was available on site, or in an online breakout room during workshops. End of workshops feedback forms asked specifically if young people needed further support from the research team or from Harmless CIC and were reminded to utilise their wellbeing plans, an overview of which can be seen in Table 5.

**TABLE 5:** Feedback.

First Workshops					
		Quantitative Feedback		Qualitative feedback	
	Total	Distress?	Support?	Positive	Negative
**Workshop 1 (online)**	*n* = 2 (66%)	*N* = 0*	*N* = 0*	Interesting, insightful, engaging, reflective, collaborative, flexible, fun activities	Wanting to know the researchers' thoughts on where the app should go
**Workshop 1 (in‐person)**	*N* = 6 (100%)	*N* = 0	*N* = 0	Good, fun, being with others, relaxed	Getting lost
**Workshop 2**	*N* = 6 (100%)	*N* = 0	*N* = 0	Being with others, interesting, meaningful, engaging activities, interactive platform	Less engaging than in‐person
**Workshop 3**	*N* = 7 (100%)	*N* = 0	*N* = 0	Meaningful, insightful, informative, enjoyable, wonderful, structured, interactive, able to influence future clinical products, progress, activities	

Second Workshops					
		Quantitative feedback		Qualitative feedback	
	Total	Distress?	Support?	Positive	Negative
**Workshop 1**	*N* = 2 (29%)	*N* = 0*	*N* = 0*	Team is kind, feel supported, grateful to be involved	
**Workshop 2**	*N* = 3 (50%)	*N* = 0	*N* = 0	Nice to see the prototype, insightful, entertaining, good interaction, informative, first hand experience	
**Workshop 3**	*N* = 3 (60%)	*N* = 0	*N* = 0	Looking forward to showcase, fun, party was great	

*Note:* *One young person left the session early because they found it overwhelming. This young person was contacted by the researchers and Harmless to ensure their safety. They did not request any further support.

Online forms collected feedback at the end of each workshop and at the end of the series of workshops to understand whether improvements could be made. Payment was provided to all young people who attended workshops at the NIHR recommended amount of £25 per hour.

Recommendations from the workshops were analysed with a member of the PPI group and written into reader friendly reports. Young people were invited to review Key Learnings and suggest changes to these reports offline and at an online ‘Findings Showcase’ event.

### Design and Development phase

#### Coproduction Group workshops

In Summer 2024, all 11 young people were invited to take part in three further coproduction workshops held online. Seven young people rejoined the next stage of workshops. In total four woman, three men took part, aged 18–25 (mean = 20.1 years), four were white British, one was black British, one was black African and one was Asian Korean. Education levels varied from A‐level to Undergraduate Degree.

In this second round of workshops, we built on previous discussions and recommendations from our Discovery work, but now focussing on the design and delivery of a prototype CaTS‐App tool. This development work covered: the content and process of completing a CaTS task using the app with a professional (workshop 1); user‐experience, screen mock‐up reviews and early prototype testing (workshop 2); and discussion around protections, privacy and outputs from the app (workshop 3).

The involvement process followed the same structure as the PPI process outlined above and detailed in Figure 2.

#### Learnings from our case‐study

**FIGURE 9:**
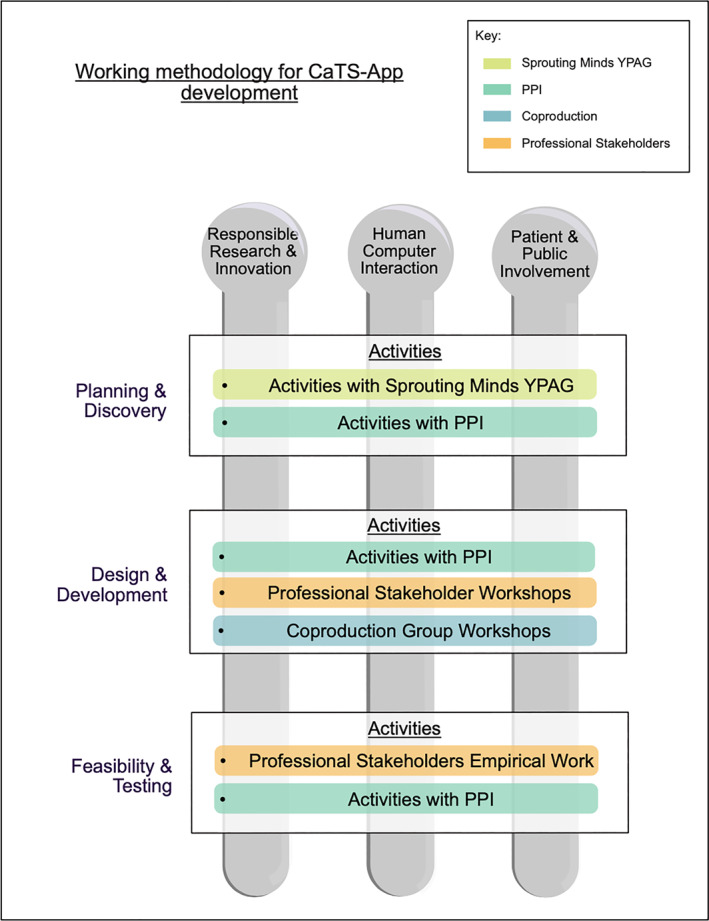
This figure includes the working methodology for the CaTS‐App development. The working methodology embeds principles relevant to user experience from PPI, HCI and RRI across all participatory stages of the codevelopment of the CaTS‐App Planning phase/Discover phase and discover phase is to build foundational understanding (via PPI and wide stakeholder research with end‐users, and coproduction workshops with young people with lived experience). Design and development phase will use co‐creation processes to produce a prototype through stakeholder workshops. Feasibility of prototype evaluated by young people and practitioner dyads to understand what worked well and what changes would be desired. This would lead to a formal feasibility evaluation study/trial to assess effectiveness.

To support understanding of how we have been, and others may continue to be guided by existing and emerging principles and models of working relevant to user experience from PPI, HCI and RRI disciplines and adopted in the development of DMHI for self‐harm, we share a summary diagram which shows how we aimed to bring together principles in the context of an app development for self‐harm (see Figure 9).

We sought to develop activities and processes which would help us to deliver involvement/coproduction with young people in the early participatory phases of our work (Planning and Discovery, and Design and Development of the CaTS‐App) in line with 4 key objectives (Table 4): Information and Clarity; Ways of Working; Safety; Recognition. Did we meet these aims? Broadly, yes though with some points for reflection.

We were able to ensure early, sustained involvement of young people from initial conception of the research project, through our Sprouting Minds co‐chairs and wider YPAG offered by the wider structure of Sprouting Minds (which also had internal support in the form of dedicated PPI managers), as well as comprehensive involvement of our dedicated PPI sub‐group. Yet, a nuance of involving young people in longer term projects is that they inevitable age, which means we need to recruit new, younger members to maintain a representative sample. Interestingly, once young people become skilled there runs the danger that they represent a different, more professionalised young person, no longer reflecting the group they initially represented (Ives et al., 2013). Therefore, the involvement of externally recruited young people for the coproduction workshops helped to ensure perspectives were not influenced by previous involvement. Further, supporting former members (both from PPI and Coproduction Groups) to transition to other roles, such as involvement within Sprouting Minds for external members of into mentoring roles for PPI members means we can retain their skills and knowledge, albeit recognising this will bring with it additional costs and resources. Unsurprisingly, given our work with young people who at times may be considered vulnerable, the active involvement of our PPI group has not always been consistent where time has been needed away from the project. This is an inevitable part of involvement in complex mental health research which should be anticipated when planning for such activities.

Taking on Sprouting Mind's mantra that ‘no one is hard to reach, it's how hard you try to reach them’, we were keen to capture voices not typically heard and marginalised groups not always represented in involvement (Hoddinott et al., 2018). We worked incredibly hard to bring together a diverse group of young people including those new to research and involvement. This took considerable time and effort but was successful largely due to taking collaborations with local community groups (see O’Hara and Lawton (2016) who advocate that researchers need to get better at engaging with different sectors of society). We believe payment supported diversity, offering an attractive renumeration for young people's contributions. Being transparent about the amount and form of money on project adverts was encouraged by our PPI group. We are fortunate in this project to be able to access ringfenced PPI budget and resources, but this is not typical or always possible for other research projects (Bélisle‐Pipon et al., 2018).

We spent time planning and developing innovative, creative methods that were interactive and fun such as collaborative whiteboards, the use of various methods to engage with activities such as emojis, scales or voting or well‐presented workshops with colourful spaces and engaging images, an investment that results in improved levels of engagement and retention (Robinson, Bailey, et al., 2018). The approach we take to working with young people could be considered strengths‐based, which utilises positive psychology over a deficit‐approach to provide agency and empowerment, focussing on solutions over problems (Russell‐Bennett et al., 2023). This raises an important question about the skills and requirements expected of researchers who deliver involvement. Failure to recognise the skills and commitment required for such task's risks devaluing the process of involvement and where necessary, collaboration across disciplines should be used to support gaps where such skillsets are not available. It can't be overstated that effective involvement in self‐harm research and involvement is hard work and requires skills, sustained commitment, resources and institutional backing and doing it well has a considerable impact on researcher workloads (Oliver et al., 2019).

The ultimate end‐goal for participatory approaches is to improve the outcome of the intervention, but to know whether the methods we use to engage young people are effective to this end requires incorporating evaluations and reflections into our processes (Orlowski et al., 2015). Charting these contributions will improve perceptions and value of PPI as an activity worthy of funding. Typically, it can be challenging to publish PPI work, yet through publication of these processes we have an opportunity to advance the field (Orlowski et al., 2015). Publications such as this, and others who have shared their process are invaluable to chart the contribution of involvement to improved outcomes. Although we have included some reflection and feedback opportunities, we aim to continue to learn and develop our evaluation measures in future involvement activities (Gan et al., 2023; Han et al., 2019; Kruzan et al., 2021).

Here, we have contributed to an established body of work which shows it is possible to safely involve young people in self‐harm research and involvement (Michail et al., 2023; Robinson, Bailey, et al., 2018) and have shown this can be achieved over a sustained period of time. Sharing where practice is working is especially important with involvement work in the UK which is not subject to formal ethical committee approvals (Hoddinott et al., 2018; Parkinson et al., 2021). The inclusion of an RRI framework provided an additional layer of structure that mandated consideration of risks and mitigation for any unintended consequences. Engaging in work in this field means a need to be able to sit with a level of risk and put in place proportional safeguards (Michail et al., 2023). Nonetheless, we recognise that our exclusion criteria preclude those at higher risk (i.e. those who have self‐harmed within 2 weeks or in crisis) from taking part, yet these young people may be the ones who would benefit most and be most representative of future users of the intervention.

## CONCLUSIONS

We have shared a working example of how we have incorporated youth involvement and coproduction into the development of the CaTS‐App and discussed ways in which our approach has been guided by existing and emerging principles and models of working relevant to user experience from PPI, HCI and RRI disciplines.

We mapped our approach to PPI and to coproduction with young people across four objectives (information and clarity, ways of working, safety, recognition) and demonstrated with illustrative examples how meeting these objectives has supported the delivery of meaningful, safe and responsible collaboration with young stakeholders. We have also illustrated a breadth of activities across the participatory phases of the app development.

These participatory phases are resource and time‐intensive processes, requiring wholesale commitment and energies from both researchers and stakeholders. Yet, the importance of user input is critical to developing interventions that are safe, effective, acceptable and implementable. It is hoped that by sharing how we have carried out involvement at an early stage of our research will stimulate further conversations in this field.

## AUTHOR CONTRIBUTIONS

**Camilla M. Babbage**: Conceptualization; data curation; formal analysis; funding acquisition; investigation; methodology; project administration; resources; software; supervision; validation; visualization; writing ‐ original draft; writing ‐ review and editing. **Joanna Lockwood:** Conceptualization; data curation; formal analysis; funding acquisition; investigation; methodology; project administration; resources; software; supervision; validation; visualization; writing ‐ original draft; writing ‐ review and editing. **Lily Roberts**: Data curation; investigation; methodology; project administration; software; visualization; writing ‐ original draft; writing ‐ review and editing. **Chris Greenhalgh:** Conceptualization; methodology; writing ‐ review and editing. **Josimar Mendes:** Conceptualization; methodology; writing ‐ review and editing. **Lucy‐Paige Willingham**: Writing ‐ review and editing. **Emmanuel Wokomah**: Methodology; writing ‐ review and editing. **Rebecca Woodcock:** Investigation; methodology; project administration; writing ‐ review and editing. **Petr Slovak:** Conceptualization; methodology; writing ‐ review and editing. **Ellen Townsend:** Conceptualization; funding acquisition; investigation; methodology; supervision; writing ‐ review and editing.

## CONFLICT OF INTEREST STATEMENT

None to declare.

## ETHICAL CONSIDERATIONS

An ethical approval number is not given as data is not presented in this article.

## Data Availability

Data sharing is not applicable to this article as no new data were created or analysed in this study.
