# Correction to “Cultivating participatory processes in self‐harm app development: A case‐study and working methodology”

**DOI:** 10.1002/jcv2.70078

**Published:** 2025-12-04

Babbage, C. M., Lockwood, J., Roberts, L., Mendes, J., Greenhalgh, C., Willingham, L.‐P., Wokomah, E., Woodcock, R., Slovak, P., & Townsend, E. (2024). Cultivating participatory processes in self‐harm app development: A case‐study and working methodology. JCPP Advances, 4(4), e12295. https://doi.org/10.1002/jcv2.12295.

In the Acknowledgements, Emma Nielsen was not included in the list of contributors associated with Digital Youth. The Acknowledgements should have been:

The contributors associated with Digital Youth With Sprouting Minds are as follows: Ellen Townsend; Chris Hollis; Jo Gregory; Elvira Perez Vallejos; Rebecca Woodcock; Peter Fonagy; Louise Arseneault; Sarah Doherty; Lucy‐Paige Willingham; Cathy Creswell; Emily Lloyd; Josimar De Alcantara Mendes; Carolyn Ten Holter; Marina Jirotka; Praveetha Patalay; Yvonne Kelly; Aaron Kandola; Edmund Sonuga‐Barke; Sonia Livingstone; Kasia Kostryka‐Allchorne; Mariya Stoilova; Rory O'Connor; Dorothee Auer; Sieun Lee; Nitish Jawahar; Marianne Etherson; Chris Greenhalgh; Kapil Sayal; Jim Warren; Vajisha Wanniarachchi; Paul Stallard; Charlotte Hall; Mathĳs Lucassen; Sally Merry; Karolina Stasiak; Camilla Babbage; Adam Parker; Holly Griffiths; Lily Roberts; Petr Slovak; Amy Jess Williams; Joanna Lockwood; **Emma Nielsen**.

In the “Second Workshops” section of Table 3, there are errors in the Gender column for Workshops 2 and 3. For Workshop 2, it should have been: Female = 4, and Male = 2. For Workshop 3, it should have been: Female = 3 and Male = 2. The correct table is:

**TABLE 3:** Coproduction workshops demography.

Second workshops				
		Demography		
	Total	Age	Gender	Ethnicity
Workshop 1	*N* = 7	18–25 (mean = 20.1)	Female = 4 Male = 3	White British = 4 Black British = 1 Asian Korean = 1 Black African = 1
Workshop 2	*N* = 6	18–25 (mean = 20.3)	**Female = 4** **Male = 2**	White British = 4 Asian Korean = 1 Black African = 1
Workshop 3	*N* = 5	18–25 (mean = 21.0)	**Female = 3** **Male = 2**	White British = 3 Asian Korean = 1 Black African = 1

We apologize for the errors.

